# Significance of case reports: Few facts more

**DOI:** 10.4103/0301-4738.77034

**Published:** 2011

**Authors:** Manoj Gautam, N S Muralidhar, Hemanthmurthy

**Affiliations:** Retina Institute of Karnatka, 122, 5^th^ Main Road, Chamrajpet, Bangalore - 560 018, Karnataka, India

Dear Editor,

We read with great interest the article entitled “The significance of case reports in biomedical publication” by Nayak.[1] We congratulate the author for an excellent description of a very relevant issue. In addition to many important points highlighted by the author, we would like to add three more points. First of all, these reports save a lot of patients from the burden of unnecessary investigations and empirical treatment, by making diagnoses in unusual clinical presentations. Secondly, these reports definitely add more to the spectrum of the disease, which results in better understanding of the natural progression of the disease along with its variations. Better knowledge transforms into better patient care, which is our primary goal. Thirdly, for the beginners, it gives exposure to scientific writing and an opportunity to get chief authorship. Otherwise, in spite of doing the major part of the work and hard labor, seldom do they get a chance for chief authorship.

In conclusion, we recommend publication of all unusual/unique/rare presentations of diseases, especially by beginners.
